# DOK7 congenital myasthenic syndrome: case series and review of literature

**DOI:** 10.1186/s12883-024-03713-0

**Published:** 2024-06-21

**Authors:** Bentolhoda Ziaadini, Bardyia Ghaderi Yazdi, Elham Dirandeh, Reza Boostani, Narges Karimi, Akram Panahi, Ariana Kariminejad, Mahsa Fadaee, Fatemeh Ahangari, Shahriar Nafissi

**Affiliations:** 1 Neurology Research Center, Kerman University of Medical Sciences, Kerman, Iran; 2 Neuromuscular Research Center, Shariati Hospital, Tehran University of Medical Sciences, Shariati Hospital, North Karegar St, Tehran, 14117-13135 Iran; 3 Department of Neurology, Shariati Hospital, Tehran University of Medical Sciences, Tehran, Iran; 4 Clinical Research Development Unit, Kowsar Educational, Research and Therapeutic Hospital, Semnan University of Medical Sciences, Semnan, Iran; 5 Department of Neurology, Mashhad University of Medical Sciences, Mashhad, Iran; 6 Department of Neurology, School of Medicine, Immunogenetics Research Center, Toxoplasmosis Research Center, Clinical Research Development Unit of Bou Ali Sina Hospital, Mazandaran University of Medical Sciences, Sari, Iran; 7 Kariminejad-Najmabadi Pathology & Genetics Center, Tehran, Iran

**Keywords:** DOK7, Whole exome sequencing, Genetic disorders, Salbutamol, Congenital myasthenic syndromes

## Abstract

**Background:**

Congenital myasthenic syndromes (CMS) are among the most challenging differential diagnoses in the neuromuscular domain, consisting of diverse genotypes and phenotypes. A mutation in the Docking Protein 7 (Dok-7) is a common cause of CMS. DOK7 CMS requires different treatment than other CMS types. Regarding DOK7’s special considerations and challenges ahead of neurologists, we describe seven DOK7 patients and evaluate their response to treatment.

**Methods:**

The authors visited these patients in the neuromuscular clinics of Tehran and Kerman Universities of Medical Sciences Hospitals. They diagnosed these patients based on clinical findings and neurophysiological studies, which Whole Exome Sequencing confirmed. For each patient, we tried unique medications and recorded the clinical response.

**Results:**

The symptoms started from birth to as late as the age of 33, with the mean age of onset being 12.5. Common symptoms were: Limb-girdle weakness in 6, fluctuating symptoms in 5, ptosis in 4, bifacial weakness in 3, reduced extraocular movement in 3, bulbar symptoms in 2 and dyspnea in 2 3-Hz RNS was decremental in 5 out of 6 patients. Salbutamol was the most effective. c.1124_1127dupTGCC is the most common variant; three patients had this variant.

**Conclusion:**

We strongly recommend that neurologists consider CMS in patients with these symptoms and a similar familial history. We recommend prescribing salbutamol as the first-choice treatment option for DOK7 patients.

## Introduction

Congenital myasthenic syndromes (CMS) do not always follow a straightforward neurophysiological presentation. This heterogeneous manifestation often results in misdiagnosis as nonspecific myopathies, therefore missing treatment, making it one of the most challenging differential diagnoses in the neuromuscular domain [1]. CMSs consist of disorders with diverse genotypes and phenotypes. The hallmark of CMS is abnormal neuromuscular transmission [1–3]. Most CMSs follow an autosomal recessive inheritance pattern. However, exceptions, such as Slow channel syndrome, SNAP25B, and SYT2, are inherited dominantly [4, 5]. 

A mutation in the Downstream of tyrosine kinase seven, Docking Protein 7 (Dok-7), is a common cause of CMS [3, 4]. It is a post-synaptic protein. To properly function, protein Dok-7 must phosphorylate Muscle-specific tyrosine kinase (MuSK) protein. It is essential for Rapsyn-associated endplate clustering of nicotinic acetylcholine receptor (AChR) [1, 4, 6, 7]. Therefore, loss of function of the *DOK7* gene causes malformation in motor endplate synapses. Many mechanisms may lead to this defect, including skipping, missense, and frameshift mutations in the *DOK7* gene [6, 8]. It usually manifests in infancy to early childhood [9]. The distinctive pattern of DOK7 CMS is a limb girdle phenotype [1, 4]. Stridor and impaired feeding are valuable diagnostic clues for early diagnosis in newborns [9]. Ptosis, facial, and neck weakness are common symptoms [4]. Patients mostly have typical milestones but often follow a worsening pattern throughout their lifetime [9]. Daily fluctuation is less evident than myasthenia gravis (MG). DOK7 has one of the worst prognoses of CMS [3]. 

Repetitive nerve stimulation (RNS) is usually decremental in CMS, and single fiber electromyography (SFEMG) yields increased jitter and blocking. Muscle biopsy is abnormal, but it is nonspecific [1, 4]. Diagnosis is confirmed by genetic testing [1, 2]. Knowing the defective protein/gene is essential in CMS because some drugs that are effective in one type may be harmful to the other [2]. DOK7 CMS requires different treatment than other CMS types, and patients generally respond very well to oral salbutamol [4, 10]. Regarding DOK7 special considerations and challenges ahead of neurologists, we describe seven DOK7 patients and evaluate their response to treatment.

## Patients and methods

### Patients

In this study, we entered seven patients diagnosed with CMS. The authors have visited these patients in the neuromuscular clinic of Shariati Hospital, Tehran University of Medical Sciences, and Kerman University of Medical Sciences Hospital.

### Methods

The authors diagnosed these patients based on clinical findings suggestive of neuromuscular junction disorder, supported by neurophysiological studies (RNS, single fiber EMG). Our patients were seronegative for anti-AChR and anti-MuSK antibodies (Ab).

According to practical electrodiagnostic protocols [11], we did a nerve conduction study including 3 Hz RNS, repetitive CMAP, and electromyography (NCS-EMG) for every patient except patient number seven. We maintained the skin’s temperature between 32 °C and 36 °C for each patient. We used Nicolet EDX (Synergy version 22.0.2.146 software, 2013 Natus) for all participants.

All of our patients had undergone Whole Exome Sequencing (WES). We considered the diagnosis of DOK7 CMS if the genetic testing revealed pathogenic or likely pathogenic variants of the *DOK7* gene.

Probands’ genomic DNA was extracted, and sequencing was performed using exome protocols. Total genomic DNA was enriched using the Agilent Human All Exon kit V6 (Agilent Technologies, Inc., Santa Clara, CA, USA) or Twist Human Core Exome (Twist Bioscience, San Francisco, CA, USA).

Subsequently, Paired-end sequencing was performed on Illumina sequencers (HiSeq 4000 and NovaSeq 6000) (Illumina, San Diego, CA, USA) according to the manufacturer’s protocol. Sequences were aligned to GRCh37/hg19 human reference sequence and variants identified through the GATK pipeline. Variations were annotated with in-house software. Common variants (≥ 1% in the general population) and recurrent artifact variant calls were filtered out based on the latest available versions of 1000G (http://www.1000genomes.org), the Exome Variant Server (http://evs.gs.washington.edu), the Exome Aggregation Consortium database (EXAC) (http://exac.broadinstitute.org), the Genome Aggregation Database (gnomAD) (https://gnomad.broadinstitute.org), Iranome (http://www.iranome.ir) and internal databases. The variant analysis emphasized the variants within genes involved in Muscular dystrophy and Muscle disorders. The final candidate variants were classified according to the American College of Medical Genetics and Genomics/Association for Molecular Pathology (ACMG/AMP) guidelines. Sanger sequencing was applied to confirm the co-segregation of variants in parents.

### Treatment

We tried unique medications for each patient and recorded the clinical response. Drugs chosen for the trial included pyridostigmine bromide, salbutamol, ephedrine, and 3,4-diaminopyridine (3,4-DAP). The authors prescribed all medicines as oral tablets. The authors assessed the patients by clinical examination and their symptoms in the follow-up visit after one month. Also, we assessed myasthenia gravis activities of daily living profile (MG-ADL) before initiating the drug trial and after showing a response a few months later.

### Results

**Fig. 1:**
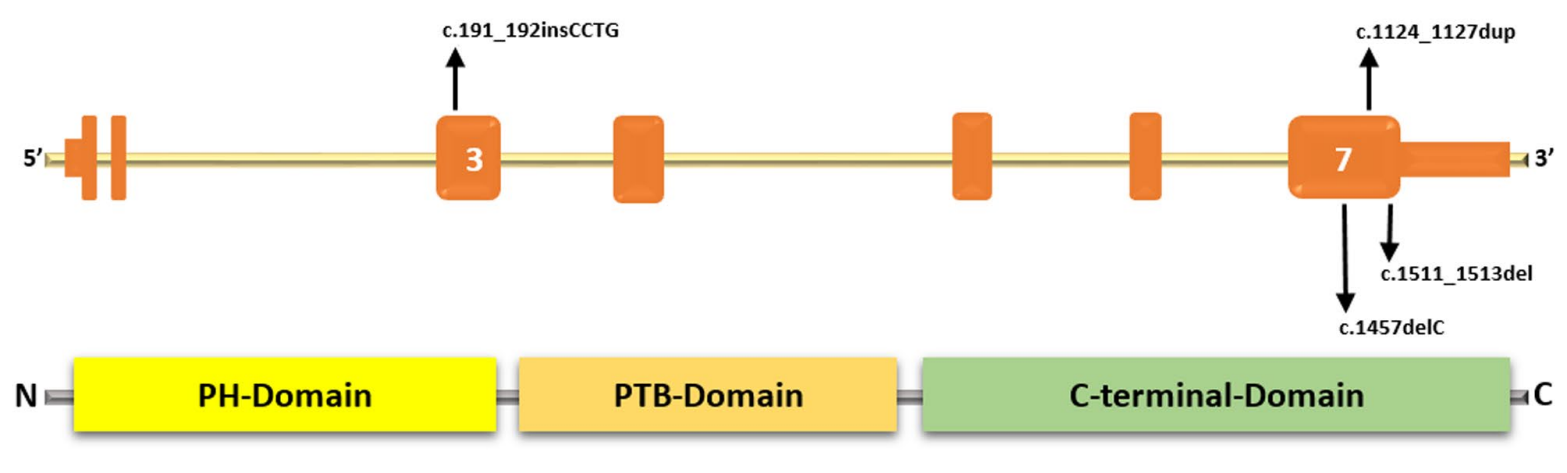
Structure of *DOK7* gene, docking protein 7, and the location of identified variations in patients of the present study

We introduce seven patients (three female and three male) with a mean age of 37.3 (from 9 to 56) (Table 1). We observed different variants in the *DOK7* gene (NM_173660.5), one novel (Table 1; Fig. 1). 3-Hz RNS was decremental in 5 patients. The mean MG-ADL before treatment was 6.16; after treatment, it was 2.66. All patients in our study showed favorable responses to salbutamol intake. After showing a favorable response to salbutamol, we discontinued pyridostigmine, considering its long-term detrimental effects. We have depicted clinical features in Tables 1 and 2. We explain the details of each patient below:

**Table 1:** Demographic data, first physical examination, and diagnostic and therapeutic findings

	Age (years)	Gender	Age of onset (years)	Duration	Consanguinity	Familial history	Dyspnea	Delayed millstone	MG-ADL	Fluctuation	Mutation	CK level (U/L)	RNS	MG-ADL after treatment	MG-ADL difference	Effective drug(s)	ineffective/deleterious drug(s)
1	9	F	3	6	+	-	-	-	4	+	c.1457delC (p.Pro486Argfs*15)	48	+	1	3	Salbutamol	Pyridostigmine
2	30	M	0	30	+	-	+	-	5	+	c.1511_1513delCTT (p.Pro504Argext*182)	160	+	3	2	Pyridostigmine3,4-diaminopyridine Salbutamol	N/A
3	56	M	16	40	+	+	-	-	4	+	C.1124_1127dupTGCC (p.Ala378Serfs*30)	150	+	1	3	Salbutamol Ephedrine	N/A
4	35	M	23	12	+	+	-	-	5	+	C.1124_1127dupTGCC (p.Ala378Serfs*30)	461	+	1	4	Salbutamol	Pyridostigmine
5	46	F	0	46	+	+	+	+	14	-	C.1124_1127dupTGCC (p.Ala378Serfs*30)	100	-	10	4	Salbutamol	Pyridostigmine
6	48	F	33	15	+	+	-	-	5	-	c.191_192insCCTG (p.Glu66Alafs*28)	1280, 480, 156	+	0	5	Salbutamol	Pyridostigmine
7	35	M	20	15	+	+	-	-	6	+	c.191_192insCCTG (p.Glu66Alafs*28)	Normal	N/A	1	5	Salbutamol	N/A

**Table 2:** Comparison of studies reporting patients with DOK7 congenital myasthenic syndrome

Studies	# cases and gender	Age at the diagnosis/age at onset (years)	Consanguinity/family history/progression	Decreased fetal movement /delayed milestone/kyphoscoliosis or lordosis/ contracture	Ptosis/ophthamoparesis or diplopia/bulbar symptoms/facial weakness	Hypotonia/neck w/proximal w/distal w/axial w/abnormal tendon reflexes	Respiratory symptoms/swallowing difficulties /stridor/ vocal cord palsy	elevated CK level/ positive rns/ repetitive cmap scan	Response to pyridostigmine/ salbutamol/ephedrine/3,4-DAP/fluoxetine
Present study	4 M/3 F	9 to 56/NB to 33	7/5/3	0/1/0/0	4/3/2/3	NA/1/6/2/1/0	2/3/1/1	1/5/0	1/7/1/1/NA
Ben Ammar et al., 2010 [1]	8 M/7 F	8 to 69/NB to 13	1/4/NA	2/10/9/NA	5/6/9/8	4/1/15/NA/NA/NA	15/2/2/NA	2/15/NA	3/NA/1/7NA
Selcen D et al., 2008 [7]	8 M/8 F	5 to 50/NB to 5	NA/NA/NA	3/NA/NA/NA	14/6/11/13	NA/NA/16/NA/5/NA	13/NA/NA/NA	NA/16/NA	1/1/3/2/NA
Klein A et al., 2013 [9]	23	NA/NB to 3	NA/NA/13	NA/16/11/4	17/4/15/18	3/NA/23/6/NA/NA	8/16/11/7	NA/11/NA	1/18/5/1/NA
Mahjneh I et al., 2013 [38]	4 M/ 2 F	41 to 53 / NB	6/6/6	NA/6/4/4	6/NA/NA/6	6/NA/6/6/6/NA	6/6/6/NA	0/NA/NA	NA/6/NA/NA/NA
Schara U et al., 2009 [35]	6 F/ 2 M	10 to 58 / NB to 12	NA/2/NA	NA/3/NA/2	8/6/4/8	3/NA/5/5/NA/NA	5/NA/NA/NA	0/4/NA	0/NA/8/NA/NA
V Mihaylova et al., 2010 [20]	2 M/2F	NB to 6 / 12 to 20	0/2/3	NA/2/3/NA	3/1/NA/2	NA/0/4/0/4/4	1/1/NA/NA	NA/3/0	1/NA/NA/NA/2
Palace J et al., 2007 [21]	4 M/11 F	19 to 63 / NB to 6	NA/NA/8	NA/0/NA/NA	9/1/6/12	3/13/15/15/11/8	8/6/NA/NA	NA/NA/NA	1/NA/5/5/NA
Muller J S et al., 2007 [26]	6 M/8 F	2 to 53 / NB to 25	NA/NA/10	NA/1/NA/NA	12/2/7/9	4/NA/13/NA/NA/NA	10/NA/NA/NA	4/11/NA	NA/NA/NA/NA/NA
Jennifer A Anderson 2008 [36]	4 M/2 F	10 to 58 / NB to 6	0/2/3	NA/0/2/NA	5/2/2/5	1/2/6/NA/NA/1	3/3/1/0	NA/6/NA	3/NA/1/6/NA

CK: Creatine kinase; RNS: Repetitive Nerve Stimulation; CMAP: Compound Muscle Action; Potential; MG-ADL: Myasthenia Gravis Activities of Daily Living Scale; 3–4 DAP: 3,4-Diaminopyridine; M: Male; F: Female; NL: Normal; N/A: None Applicable; these studies did not report this item

### Patient #1

This nine-year-old girl has a history of generalized weakness, ptosis, and eye deviation, starting when she was three years old. Her symptoms were fluctuating. Her parents were first cousins but denied similar problems in themselves and their relatives. In the neurological examination, there was mild ptosis and mild limitation of eye movements in all directions. In addition, there was severe weakness in the proximal lower and upper limbs.

Creatinine kinase (CK) was 48 units/L (normal range: 24–195 units/L). Except for positive RNS, the rest of the electrodiagnostic (EDX) study was normal.

Whole exome sequencing revealed a Homozygous likely pathogenic variant defined as c.1457delC (p.Pro486Argfs*15) in exon 7 of the *DOK7* gene. Although resulting in a nonsense-mediated decay is not predicted, this deletion causes a frameshift starting with codon Proline 486, affecting the last 19 amino acids of the protein Dok-7. One autosomal recessive congenital myasthenic syndrome patient showed this premature translational stop signal [12]. 

The patient’s condition worsened when we prescribed pyridostigmine. Salbutamol was started, and she had significant improvement after two months. MG-ADL before introducing salbutamol was four and reached one after treatment. She did not tolerate doses above 1 mg three times a day (TDS), and she has been receiving this dose for five years.

### Patient #2

This patient is a thirty-year-old man who, as far as he remembered, had motor skills that were not similar to other children. From birth, he was short stature and had eye deviation, diplopia, and mild facial weakness. Dyspnea from birth necessitated a tracheostomy when he was thirteen years old. His symptoms were fluctuating. His parents were first cousins, and there was no record of similar medical conditions in his family members. The neurological examination confirmed ptosis, limited extraocular muscle movements, and mild facial weakness. Limb and neck muscles had normal strength.

He had a CPK level of 150 units/lit. Low-frequency RNS showed a decremental response.

WES revealed a homozygous likely pathogenic c.1511_1513delCTT (p.Pro504Argext*182) variant in exon 7 of the *DOK7* gene. This deletion causes a frameshift, disrupting the natural stop codon of the protein Dok-7 and extending the protein by 182 additional amino acid residues. This protein extension is a known culprit in individuals with congenital myasthenic syndrome [8, 13]. 

He has been on pyridostigmine since infancy. Salbutamol was started four years ago after the results of genetic testing came back. We tried to taper off pyridostigmine, but he was very dependent on this medication and did not tolerate reducing the dose. 3,4-diaminopyridine improved his condition, but he developed paresthesia and abdominal cramps. He is currently receiving pyridostigmine 60 mg four times a day (QID) and salbutamol 2 mg QID. His MG-ADL improved from 5 to 3 on these medications. He closes the tracheostomy in the daytime but has to open it at night. Otherwise, he will develop stridor.

### Patient #3

The next patient was a fifty-six-year-old man who had fatigue and difficulty changing to an upward position since the age of 15. His weakness varied in severity and worsened with stressful events. He had mild problems with emotional expression, smiling, blowing, and eating. These symptoms gradually declined during the past years. His symptoms were fluctuating. He had two sisters with similar symptoms; one of them was misdiagnosed as myasthenia gravis and died after thymus surgery at the age of 50. Mild facial weakness and severe weakness of all limbs (more prominent in proximal muscles) were evident in neurological examination. He had no ptosis or extraocular muscle weakness but had nasal speech and dysarthria. The parents were first cousins.

Laboratory data showed a normal CK. (150 units/L) EDX showed a significant decremental response with low-frequency RNS without repetitive CMAP.

Whole exome sequencing showed a homozygous pathogenic variant defined as c.1124_1127dupTGCC (p.Ala378Serfs*30) in exon 7 of the *DOK7* gene. This frameshift duplication has been commonly reported in the literature in homozygosity or compound heterozygosity with other pathogenic variants in individuals affected with DOK7 CMS patients [14]. 

He had a long history of prednisone intake for several years before the diagnosis, and this was gradually tapered and discontinued. Some of his Symptoms (limb weakness) improved with pyridostigmine, but dysarthria became worse, with incomprehensible speech. He received ephedrine for a few months, which improved lower limb function, but the arm weakness deteriorated. Improvement began after one month of salbutamol 2 mg TDS, but after three months of salbutamol and discontinuation of pyridostigmine, there was a dramatic improvement. He has been on salbutamol for ten years and only has mild arm weakness. The MG-ADL went from 4 to 1 after treatment.

### Patient #4

This patient was a thirty-five-year-old man whose problem started two years ago. Eating, drinking, and swallowing were difficult for him during these years and often caused nasal regurgitation. His symptoms were fluctuating. The parents were first cousins, and there is a history of ptosis in the mother and a brother and sister, but they have not been examined or evaluated. In the neurological examination, bilateral ptosis was visible. Movement of extraocular and facial muscles was normal. He had moderate weakness in limb muscles, but his neck and truncal muscles were normal.

CK was 461 units/L (higher than the upper limit of the normal range). RNS was positive, and there was no repetitive CMAP.

Like patient 3, this patient also had a homozygous pathogenic c.1124_1127dupTGCC variant in *DOK7*.

Because he was diagnosed with myasthenia gravis, he has received prednisone and azathioprine for a few months. Those drugs were discontinued, and he showed some improvement. Then, pyridostigmine was started, and the weakness worsened. He was improved with salbutamol 2 mg QID after two months, and the improvement was maximum after six months. He is currently receiving salbutamol 2 mg TDS, and his MG-ADL score has improved from 5 to 1.

### Patient #5

This patient was a forty-six-year-old woman. Based on her parents’ records, she has had dyspnea since birth and has used non-invasive ventilation (NIV) recently. She had delayed motor milestones. She did not have any fluctuating pattern in the symptoms, and she did not mention diplopia. She had mild difficulty in eating and swallowing. Her parents were first cousins. She noticed similar symptoms in her nephew. Neurological examination showed ptosis and limited extraocular muscle movement. In addition to bulbar symptoms and mild facial weakness, mild limb weakness and weak neck and truncal muscles were apparent.

CK was in the normal range (100 units/L). We performed complete EDX, including RNS, which was normal.

This patient had a homozygous pathogenic variant c.1124_1127dupTGCC.

As a treatment trial, we asked her to use pyridostigmine, which caused deterioration in her symptoms and complaints. Salbutamol 1 mg twice daily (BD) significantly improved her Symptoms, with an MG-ADL score change from 14 to 10 after six months. Also, she does not require NIV during the day but uses it through the night.

### Patient #6 and #7

The sixth patient was a 47-year-old woman. She has noticed lower limb weakness from 15 years ago, slowly progressing to her upper limbs. The symptoms were not fluctuating, but fatigue relatively aggravated them. As her symptoms progressed, she came to the neuromuscular clinic. Her parents are first cousins, and her nephew has shown graver signs since age twenty. The iliopsoas and quadriceps muscle forces were 1/5. The tibialis anterior muscle force was 3/5, and the deltoid and biceps muscle forces were 4/5. There was no ptosis or facial weakness. DTR was also normal.

She had three CK levels of 1280, 480, and 156 U/L. We performed complete EDX and RNS. RNS was decremental.

WES revealed a homozygous likely pathogenic variant described as c.191_192insCCTG (p.Glu66Alafs*28) in exon 3 of *DOK7*. This insertion causes a frameshift starting with codon Glutamate 66, changes this amino acid to an Alanine residue, and creates a premature stop codon at position 28 of the new reading frame. This variation is novel and has not been published as a variant, nor has it been reported as a benign polymorphism so far.

As a treatment trial, we started pyridostigmine 60 mg TDS, which aggravated her symptoms, and we changed it to salbutamol 2 mg BD. Salbutamol intake improved her MG-ADL score from 5 to 0, and all muscle forces became 5/5.

Patient number seven, the nephew of the sixth patient, is 35 and has also had fluctuating proximal weakness without cranial muscle involvement since the age of 20. His parents were first cousins and had normal CPK levels. He chose not to undergo RNS. He had the same variant as his aunt. He received salbutamol 2 mg BD after his aunt was diagnosed and had a dramatic response. MG-ADL improved from 6 to 1.

## Discussion

More than 30 identified genes cause CMS. These mutations cause synaptic, presynaptic, or post-synaptic defects [15]. Essential genes that encode components of NMJ include the genes that encode acetylcholine receptor subunits (*CHRNA1*, *CHRNB1*, *CHRND*, *CHRNE*, and *CHRNG*), choline acetyltransferase (*CHAT*), the collagen tail subunit of acetylcholinesterase (*COLQ*), rapsyn (*RAPSN*), MuSK (*MUSK*), and the skeletal muscle sodium channel NaV1.4 (*SCN4A*). Beeson et al. recognized protein Dok-7 as an essential NMJ protein in 2006, and its locus is a major locus for mutations responsible for limb-girdle type CMS [14, 16]. Protein Dok-7 impairment destroys NMJ synaptic structure [7]. DOK7 CMS is one of the most common CMS subtypes. It is reported to occur in 10-18% of CMSs [17]. The prevalence varies across geographical territories. For example, mutations in glycosylation genes *GFPT1*, *DAPG1*, and *GMPPB* are more common in India [18]. For diagnosing CMS, a *clinical tetrad* can be defined as including fatigable weakness, prominent in ocular and other cranial muscles, childhood-onset, negative myasthenia gravis autoantibodies, and supportive electrophysiological data in the form of positive slow RNS or abnormal single fiber EMG [17, 19]. 

In some studies, *DOK7* variants are the second most common mutation after *CHRNE* [20]. They were present in a large proportion (12/26, 46%) of CMS patients whose screening did not detect a mutation in other CMS genes. Thus, *DOK7* variants were present in around 12% of CMS kinships confirmed by genetic diagnosis, and *DOK7* was the third most commonly affected gene in their study. Like the other studies and our study, the authors found four nucleotide frameshift mutations c.1124_1127dupTGCC was a common variant (present in 20/24 kinships) [21]. 

Selcen et al. evaluated the phenotype and genotype of 16 patients diagnosed with DOK7 myasthenia. They gathered biopsies and conducted electromyographic studies to evaluate neuromuscular transmission. They showed that protein Dok-7 has an essential role in the size and integrity of endplate potentials, and its defect can cause variable symptoms [7]. Amongst the *DOK7* variants, c.1124_1127dupTGCC, leading to a frameshift mutation and premature termination of *DOK7*, is the most common mutation [22–24]. This suggests a founder effect with Central/Western European origin and shared ancestry in Brazil [22, 25]. In an interesting cohort, all but six of 28 DOK7 patients reported at least one copy of this frameshift mutation. They also identified five additional CMS patients who were heterozygous carriers of c.1124_1127dupTGCC. Remarkably, the patients with late-onset symptoms were all homozygous for the common variant, c.1124_1127dupTGCC [26]. In our study, the c.1124_1127dupTGCC variant is the most common; three patients had this variant. One of these patients had been symptomatic from birth. She had delayed millstone and respiratory involvement after birth.

*DOK7* variants may cause different symptoms in different stages of life [7], but they usually present as limb girdle weakness in adults [21, 27]. The course of disease is progressive throughout life [26, 28]. Six patients in our study had progressive limb girdle weakness.

Symptoms might fluctuate over more extended periods, like five of our patients. In a study, most of their DOK7 patients had ptosis, and facial weakness was frequent [26]. Three patients in our study had ptosis, and three had mild facial weakness. The mentioned study showed that many patients suffered from deterioration of respiratory function and bulbar weakness or experienced respiratory crises [26]. This was the case with two of our patients. Three of our patients had impaired eye movement. This finding is contrary to other articles [16, 17, 21, 26, 29]. Although it appears that CMS is a rare cause of congenital stridor due to bilateral vocal cord palsy [17, 29, 30], patient#2 has been suffering from stridor since infancy.

The field of CMS has rapidly grown with the deployment of next-generation sequencing [10]. Confirmatory genetic testing for CMS includes single gene analysis (guided by family history or predictive phenotype), multigene CMS panels, broader neuromuscular gene panels, whole exome sequencing, or whole genome sequencing. Multigene panels and whole exome sequencing are standard in diagnosing these conditions [11]. We discovered our patients’ variants by whole exome sequencing. CMSs are rare disorders, and only small patient numbers are available for randomized controlled trials, which causes significant limitations for treatment efficacy studies [28]. Careful selection of treatment based on WES is essential as some drugs are helpful or maybe deleterious for certain types of CMS [19]. 

There were three categories of agents used for the treatment of CMS: cholinergic agents pyridostigmine and 3,4-DAP, which increase the availability of ACh in the NMJ; the long-lived open-channel blockers fluoxetine and quinidine used in the slow-channel syndrome, as well as the β-adrenergic agonists salbutamol (albuterol) and ephedrine [19]. Salbutamol stabilizes NMJ structure by decreasing the detrimental effects of long-term acetylcholinesterase inhibitors on the post-synaptic NMJ and reducing the dispersion of the ACh receptor [19, 31, 32]. Pyridostigmine and 3,4 DAP are no longer being used routinely as treatment options for DOK7 CMS. It is believed that by enhancing neuromuscular transmission, these drugs can potentially destabilize the synapse through the ACh dispersal pathway [5]. These drugs can worsen the symptoms [12, 19, 21, 28, 29]. Considering that DOK7 patients deteriorate on pyridostigmine, the authors initiated pyridostigmine in these patients because they prescribed it before the genetic diagnosis. They discontinued pyridostigmine after observing the deteriorating effect of this medication on patients or after seeing the genetic results.

Some CMS patients improve with the sympathomimetic drug ephedrine [17, 19, 29, 30]. Endplate AChE deficiency due to defect in ColQ and Dok-7 proteins is the most prevalent CMS syndrome that responds more selectively to β-adrenergic agonists. Treatment responses are both consistent and often lead to remarkable functional improvement [19]. Some patients were wheelchair dependent before treatment but, after treatment with the drugs, could walk unaided [17]. The improvement is often seen in 1–2 months and continues for over one year [17, 33]. So β-Adrenergic agonists alone or as an add-on therapy with other CMS drugs are beneficial to many patients [12, 17, 19, 28–31, 33–36]. Oral salbutamol is also effective in AChR deficiency. It is especially effective in severe cases where patients are on long-term pyridostigmine therapy, destabilizing the post-synaptic muscle membrane [37]. 

However, ephedrine has both α and β-adrenergic effects, and concern remains related to central and cardiac adverse effects, especially with long-term use in children [28, 34]. Salbutamol, a selective β2-agonist, has been used successfully in pediatrics as a bronchodilator to treat asthma [17, 28] and more recently to improve muscle strength in Spinal Muscular Atrophy and some congenital myopathies [28]. It was influential in DOK7 CMS in 15 patients (five treated between ages 5 and 17 years) in a 2011 report from Mayo Clinic, and a few patients no longer required a wheelchair [17]. Salbutamol may have a similar benefit to ephedrine in CMS. The mentioned article offered salbutamol as first-line therapy to youths with DOK7 CMS to lower the α-adrenergic side effects [28]. Six DOK7 CMS patients responded to salbutamol in another study [38]. Salbutamol improved the condition of all seven patients in our study.

## Conclusion

In this article, we present seven patients with DOK7 congenital myasthenic syndrome, with different presentations and ages of onset. The symptoms started from birth to as late as the age of 33, with the mean age of onset being 12.5. Common symptoms were (number of patients): Limb-girdle weakness [6], fluctuating symptoms [5], ptosis [4], mild bifacial weakness [3], reduced extraocular movement [3], bulbar symptoms [2] and dyspnea [2].

3-Hz RNS was decremental in 5 out of 6 patients. There was no repetitive CMAP. Of various drugs prescribed for these patients, salbutamol was the most effective, significantly improving the MG-ADL score of all patients. Salbutamol reduced mean MG-ADL from 6.16 to 2.66. In our study, the c.1124_1127dupTGCC variant is the most common; three patients had this variant. We strongly recommend that neurologists consider CMS in patients with these symptoms and a similar familial history. We recommend the prescription of salbutamol as the first-choice treatment option for DOK7 patients. Also, we recommend waiting for genetic confirmation in patients with features suggestive of DOK7 CMS. We do not recommend trying pyridostigmine before genetic results due to its deteriorating effects and the problems that this drug generally causes for DOK7 patients.

## Data Availability

No datasets were generated or analysed during the current study.
